# Liposomal Formulations of a Polyleucine–Antigen Conjugate as Therapeutic Vaccines against Cervical Cancer

**DOI:** 10.3390/pharmaceutics15020602

**Published:** 2023-02-10

**Authors:** Farrhana Z. Firdaus, Stacey Bartlett, Waleed M. Hussein, Lantian Lu, Quentin Wright, Wenbin Huang, Ummey J. Nahar, Jieru Yang, Mattaka Khongkow, Margaret Veitch, Prashamsa Koirala, Uracha R. Ruktanonchai, Michael J. Monteiro, Jazmina L. Gonzalez Cruz, Rachel J. Stephenson, James W. Wells, Istvan Toth, Mariusz Skwarczynski

**Affiliations:** 1 School of Chemistry and Molecular Biosciences, The University of Queensland, St. Lucia, QLD 4072, Australia; 2 Faculty of Medicine, Frazer Institute, The University of Queensland, Brisbane, QLD 4102, Australia; 3 National Nanotechnology Center (NANOTEC), National Science and Technology Development Agency, 111 Thailand Science Park, Phahonyothin Rd., Khlong Luang, Pathumthani 12120, Thailand; 4 Australian Institute for Bioengineering and Nanotechnology, The University of Queensland, Brisbane, QLD 4072, Australia; 5 School of Pharmacy, The University of Queensland, Woolloongabba, QLD 4102, Australia

**Keywords:** cervical cancer, antitumor peptide vaccine, poly(amino acid), liposome, mannose-based targeting moiety

## Abstract

Human papilloma virus (HPV) is responsible for all cases of cervical cancer. While prophylactic vaccines are available, the development of peptide-based vaccines as a therapeutic strategy is still under investigation. In comparison with the traditional and currently used treatment strategies of chemotherapy and surgery, vaccination against HPV is a promising therapeutic option with fewer side effects. A peptide derived from the HPV-16 E7 protein, called 8Qm, in combination with adjuvants showed promise as a therapeutic vaccine. Here, the ability of polymerized natural amino acids to act as a self-adjuvating delivery system as a therapeutic vaccine was investigated for the first time. Thus, 8Qm was conjugated to polyleucine by standard solid-phase peptide synthesis and self-assembled into nanoparticles or incorporated in liposomes. The liposome bearing the 8Qm conjugate significantly increased mice survival and decreased tumor growth after a single immunization. Further, these liposomes eradicated seven-day-old well-established tumors in mice. Dendritic cell (DC)-targeting moieties were introduced to further enhance vaccine efficacy, and the newly designed liposomal vaccine was tested in mice bearing 11-day-old tumors. Interestingly, these DCs-targeting moieties did not significantly improve vaccine efficacy, whereas the simple liposomal formulation of 8Qm-polyleucine conjugate was still effective in tumor eradication. In summary, a peptide-based anticancer vaccine was developed that stimulated strong cellular immune responses without the help of a classical adjuvant.

## 1. Introduction

Over 600 thousand people are diagnosed with cervical cancer each year triggered by the human papilloma virus (HPV), and 342 thousand people died worldwide as a result of the disease in 2020 alone [1]. Over the past few decades, the number of HPV-related cancer cases has risen, especially in developing countries. While early identification through comprehensive cervical screening programs has decreased the prevalence of cervical cancer, these resources are less accessible in under-developed countries. Treatment options currently available for cervical cancer include chemotherapy, radiotherapy and surgery. However, these treatments have a high failure rate due to significant relapse rates and the development of drug resistance. A prophylactic vaccine has been developed against HPV infection, which will likely decrease the overall rate of cervical cancer; however, population-scale effects from this vaccine are thought to be at least 20 years away [2]. Furthermore, the risk of people already infected with HPV developing cervical cancer is high. Therefore, the development of a therapeutic vaccine that targets HPV-infected cells is important for treating established cervical cancer and other HPV-associated cancers.

Vaccines designed to treat cancer need to stimulate cytotoxic T-lymphocyte (CTL) responses in order to eliminate abnormal cells bearing specific tumor antigens. The use of whole-HPV or the HPV oncoprotein as an antigen can lead to oncogenic changes. Therefore, the development of a peptide-based vaccine has been suggested [3,4,5]. The HPV E7 oncoprotein is found in HPV-infected cells and is responsible for HPV-associated tumor cell growth; furthermore, it is the most commonly targeted antigen for therapeutic vaccine development [6,7,8,9]. **8Qm** (E744-57, QAEPDRAHYNIVTF), a peptide epitope derived from HPV-16E7 oncoprotein, which is recognized by CD4+ and CD8+ T-lymphocytes, has been shown to have a therapeutic effect against cervical cancer in mice [10,11]. Peptides alone, including **8Qm**, are not able to stimulate any significant immune response due to their poor immunogenicity and low stability in vivo. Instead, peptides need to be incorporated into a delivery system or co-administered with adjuvants to produce an effective vaccine. However, problems associated with the use of currently available adjuvants are common, including toxicity, low efficacy and a limited choice of adjuvants appropriate for human use [12,13]. Moreover, most of the clinically investigated subunit vaccines are unable to elicit strong CD8^+^ T-cell responses [14], and FDA-approved adjuvants are usually poor CD8^+^ T-cell activators [15]. Therefore, developing novel adjuvants (or delivery systems) with potent immunomodulatory effects on the cells of the innate and adaptive immune systems without adverse toxicity at therapeutic doses is of great importance in the field of cancer immunotherapy. To date, vaccines assessed in clinical trials have struggled to eliminate advanced cancers and, consequently, no clinically approved therapeutic cancer vaccines currently exist (excluding the vaccine-like dendritic-cell-based treatment sipuleucel-T [Provenge^®^] for prostate cancer) [16].

Previously, we demonstrated that **8Qm** conjugated to polyacrylates self-assembled to form microparticles. These microparticles, following a single immunization, triggered the eradication of young (three-day-old) tumors in mice [10,11,17]. However, when vaccination was delayed to seven days after tumor implantation, mouse survival rate dropped significantly, even when booster immunizations were administered [18]. Excitingly, after the **8Qm**-polyacrylate conjugate was formulated into liposomes (**L1**), the tumor eradication potency was improved, with three out of five mice being tumor-free 2 months after tumor implantation [19]. However, polyacrylates are not biodegradable, have undefined stereochemistry and contain a variable number of repeating monomer units in each polymer. Considering the shortcomings of synthetic polymers, we designed fully defined and biodegradable polymers built from natural hydrophobic amino acids (pHAAs) that can be synthesized using classic solid-phase peptide synthesis (SPPS) [20]. This method is fully automated and used to incorporate desired peptide epitopes into molecules within a single procedure. Moreover, pHAAs, such as polyleucine (**pLeu**), mimic the transmembrane fragments of proteins, which are often leucine-rich and, therefore, have been used as anchoring moieties for the incorporation of peptide antigen in liposomal delivery systems [21]. While pHAA systems are still classified as polymeric, they are tremendously different to other polymeric systems currently used for vaccine delivery [22]. The application of fully defined natural hydrophobic amino acids serving as monomers may overcome all disadvantages of classic polymer-based delivery systems. We have demonstrated that a pHAA sequence consisting of **pLeu** conjugated to the J8 B-cell epitope from group A *Streptococcus* self-assembled into nanoparticles stimulated the production of high titers of opsonic antibodies in mice [23,24]. Moreover, **pLeu**-based peptide vaccines were very effective in triggering humoral immunity against hookworm and in parasite clearance from infected animals [25,26,27]. Here, we hypothesize that **pLeu** can be also employed in a conjugation-based delivery system to induce cytotoxic T-lymphocyte (CTL) responses. Moreover, **pLeu** conjugates anchored to DC-targeting liposomes were expected to demonstrate improved anticancer efficacy. 

**Figure 1:**
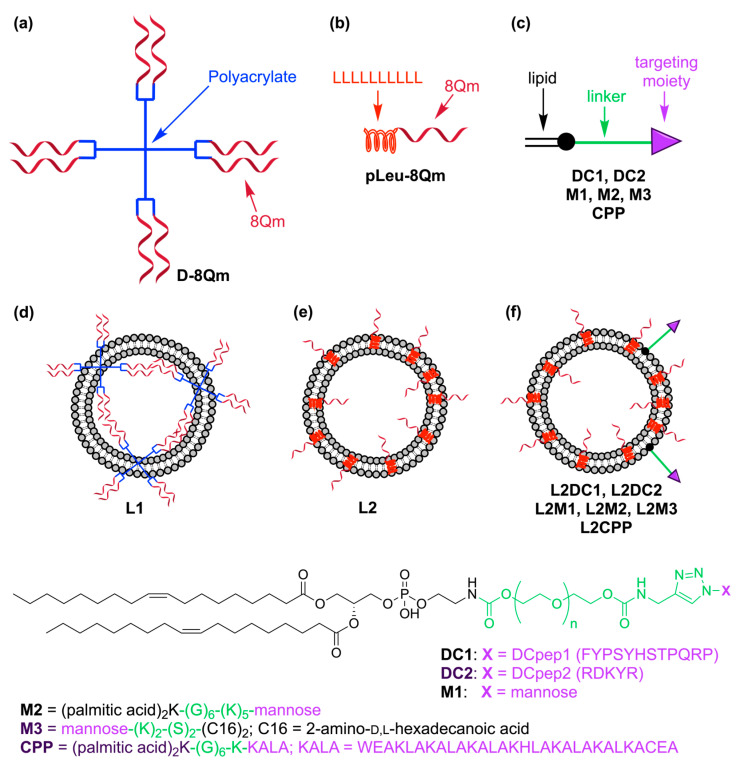
Schematic representation of the vaccine candidates used in the study: (**a**) **D-8Qm**; (**b**) **pLeu-8Qm**; (**c**) DCs-targeting moieties and liposomes; (**d**) **L1** bearing **D-8Qm**; (**e**) **L2** bearing **pLeu-8Qm**; and (**f**) **L2M** bearing **pLeu-8Qm** and targeting moieties. Peptide sequences are shown with their one-letter amino acid code.

The aim of this study was to develop a new antitumor vaccine based on a novel polymeric/liposomal delivery system and demonstrate its efficacy against cervical cancer. **pLeu** was attached to the **8Qm** peptide to form **pLeu-8Qm,** and it was then anchored into a secondary delivery system involving liposomes. A variety of dendritic cell (DC)-targeting moieties were further incorporated into these liposomes to potentially enhance the vaccine’s efficacy (Figure 1).

## 2. Materials and Methods

### 2.1. Materials

All chemical materials used in this study were analytical grade unless otherwise stated. Protected Fmoc amino acids and 1-[bis(dimethylamino)methylene]-1H-1,2,3-triazolo [4,5-b] pyridinium 3-oxide hexafluorophosphate (HATU) were purchased from Mimotopes (Melbourne, VIC, Australia). Dichloromethane (DCM), diethyl ether, piperidine, trifluoroacetic acid (TFA), N,N′-dimethylformamide (DMF), N,N′-diisopropylethylamine (DIPEA), HPLC grade acetonitrile and methanol were purchased from Merck (Darmstadt, Germany). Triisopropylsilane (TIS), acetic anhydride, erythrocyte lysing buffer, pilocarpine, phosphate-buffered saline (PBS), phenylmethyl-sulfonylfluoride (PMSF), anti-mouse IgG and IgA conjugated to horseradish peroxidase were purchased from Sigma-Aldrich (St. Louis, MO, USA). IC fixation buffer and phenol-free IMDM Glutamax medium were obtained from Gibco® Life Technologies (Carlsbad, CA, USA). Dipalmitoylphosphatidylcholine (DPPC), cholesterol, dimethyldioctadecylammonium bromide (DDAB), Avanti mini extruder, PC membranes and filter supports were bought from Avantis Polar, Inc. (Auspep Pty Ltd., Tullamarine, VIC, Australia). Copper wires were purchased from Aldrich (Steinheim, Germany). All other reagents were obtained at the highest available purity from Sigma-Aldrich (Castle Hill, NSW, Australia). 

ESI-MS was performed using a Perkin Elmer Sciex API 3000 instrument with Analyst 1.4 software (Applied Biosystems/MDS Sciex, Toronto, ON, Canada). Analytical RP-HPLC was performed using Shimadzu (Kyoto, Japan) instrumentation (DGU-20A5, LC-20AB, SIL-20ACHT, SPD-M10AVP) with a 1 mL/min flow rate and detection at 214 nm and/or an evaporative light-scattering detector (ELSD). Separation was achieved using a 0–100% linear gradient of solvent B over 40 min on either a Vydac analytical C4 column (214TP54; 5 μm, 4.6 mm × 250 mm) or a Vydac analytical C18 column (218TP54; 5 μm, 4.6 mm × 250 mm), where solvent A was 0.1% TFA/H_2_O and solvent B was 90% MeCN/0.1% TFA/H_2_O. Preparative RP-HPLC was performed on Shimadzu (Kyoto, Japan) instrumentation (either LC-20AT, SIL-10A, CBM-20A, SPD-20AV, FRC-10A or LC-20AP × 2, CBM-20A, SPD-20A, FRC-10A) in linear gradient mode using a 5–20 mL/min flow rate with detection at 230 nm. Separations were performed with solvent A and solvent B on a Vydac preparative C18 column (218TP1022; 10 μm, 22 mm × 250 mm), Vydac semi-preparative C18 column (218TP510; 5 μm, 10 mm × 250 mm) or Vydac semi-preparative C4 column (214TP510; 5 μm, 10 mm × 250 mm). The particle size distribution and measurement of the average particle size were analyzed by dynamic light scattering (DLS) (Malvern Instruments, Malvern, UK). Multiplicate measurements were taken, and the average particle size calculated. MALDI-TOF mass spectrometry samples (1 mg/mL) were spotted onto a MALDI plate with α-cyano-4-hydroxycinnamic acid matrix. A blend of peptide and protein calibrants (Bruker, Billerica, MA, USA) was used for external calibration. MALDI-TOF MS was acquired on a Bruker Autoflex III in negative modes (linear) across a mass range of 2000–10,000 *m*/*z*, with spectra gated to 2000 *m*/*z*.

### 2.2. Synthesis of Peptides, Conjugates and Targeting Moieties

**D-8Qm** and **8Qm** were synthesized as reported previously [19].

**Synthesis of pLeu-8Qm**

**pLeu-8Qm** was synthesized by manual stepwise SPPS on rink amide MBHA resin (substitution ratio: 0.52 mmol/g, 0.1 mmol scale, 0.192 g) using HATU/DIPEA Fmoc-chemistry according to the standard procedure [28]. The product was purified using an RP-HPLC C4 column with a 50–80% solvent B gradient over 30 min. tR = 32.0 min, purity >95%. Yield: 32%. ESI-MS: *m*/*z* 1417.7 (calculated 1417.2) [M+2H]^2+^; 945.7 (calculated 945.2) [M+3H]^3+^; MW = 2832.48 (Supporting Information, Figure S1).

**Synthesis of DC1**

DOPE-PEG3.4k-alkyne (Supporting Information, Figure S2) was synthesized in the same manner as we previously reported [29]. Azido-acetic-acid-modified peptides DCpep1 (N3CH2C(O)-FYPSYHSTPQRP) and DCpep2 (N3CH2C(O)-RDKYR) were synthesized using a standard protocol [28]. The peptides were individually conjugated to compound **4** using copper (I)-catalyzed alkyne azide 1,3-dipolar cycloaddition (CuAAC) following our standard method [30]. Namely, azido-acetic-acid-modified DCpep1 (3.7 mg, 2.4 μmol, 2.4 equiv.) and DOPE-PEG3.4k-alkyne (4.2 mg, 1.0 μmol, 1.0 equiv.) were dissolved in DMF (1 mL) and added to the pre-washed and dried copper wires (60 mg). The reaction mixture was degassed with nitrogen and stirred for three hours at 50 °C in the dark under an atmosphere of nitrogen. The copper wire was removed by filtration and washed using DMF (0.5 mL) before the reaction solution was slowly added (0.005 mL/min) to water (3 mL) to form micelles by the self-assembly process. The formed particles were extensively dialyzed against water in a 2 kDa dialysis bag for three days. Following lyophilization, compound **DC1** was formed as a white powder and characterized using MALDI-TOF mass spectrometry. Compound **DC1** yield: 71%. Purity: 99%. Molecular Weight: 5778. MALDI-TOF: MW_calculated average_ = 5779; MW_found_ = 5780 (Supporting Information, Figure S3). 

**Synthesis of DC2**

Azido-acetic-acid-modified DCpep2 (3.3 mg, 3.6 μmol, 4 equiv.) and DOPE-PEG3.4k-alkyne (3.8 mg, 0.9 μmol, 1.0 equiv.) were dissolved in DMF (1 mL). The rest of the **DC2** synthesis was carried out in an identical manner to that described for the synthesis of **DC1**. Yield: 63%. Compound **DC2** yield: 63%. Purity: 99%. Molecular Weight: 5134. MALDI-TOF: MW_calculated average_ = 5135; MW_found_ = 5136 (Supporting Information, Figure S3).

**Synthesis of M1**

DOPE-PEG3.4k-alkyne was conjugated to mannose azide [31] using CuAAC reaction following our standard method [30]. The production of **M1** was confirmed with MALDI-TOF analysis. Compound **M1** yield: 37%. Purity: 99%. Molecular Weight: 4509. MALDI-TOF: MW_calculated average_ = 4510; MW_found_ = 4502 (Supporting Information, Figure S4).

**Synthesis of M2, M3 and CPP**

Synthesis of these lipopeptides was performed as reported [31,32].

### 2.3. Formulation of Vaccine Candidates

**Formulation of L1**

**D-8Qm** and liposomes **L1** were formulated as previously reported [19]. 

**Formulation of pLeu-8Qm**

**pLeu-8Qm** was dissolved in Millipore endotoxin-free water (900 μL) and self-assembled into nanoparticles. Prior to injection, PBS (100 μL, 10×) was added to the formulation, producing the final concentration of 1 mg/mL.

**Formulation of L2**

Dipalmitoylphosphatidylcholine (DPPC), didodecyldimethylammonium bromide (DDAB) and cholesterol were each dissolved in chloroform (1 mL) to achieve final concentrations of 10 mg/mL, 10 mg/mL and 5 mg/mL, respectively. Solutions of DPPC (0.5 mL), DDAB (0.2 mL) and cholesterol (0.2 mL) were added to a round-bottom flask containing chloroform (2 mL). **pLeu-8Q** (1 mg) was dissolved in methanol (1 mL) and added to the flask. The solvents were then slowly evaporated under reduced pressure using a rotatory evaporator before the residual solvent was removed under vacuum overnight generating a lipid film. The lipid film was rehydrated with Millipore endotoxin-free water (900 μL) at 56 °C following extrusion with 200 nm pore-size polycarbonate membrane. Prior to injection, PBS (100 μL, 10×) was added to the formulation, producing the final concentration of **pLeu-8Qm** (1 mg/mL) in **L2**. The encapsulation efficiency of **pLeu-8Qm** in liposomes was 99%. Encapsulation was determined as previously reported [19].

**Formulation of L2M1, L2M2, L2M3, L2DC1 and L2DC2**

Liposomes bearing targeting moieties were formulated in a similar manner to **L2**, except DC-targeting moieties **M1** (0.233 mg), **M2** (0.063 mg), **M3** (0.098 mg), **DC1** (0.299 mg), **DC2** (0.265 mg) and **CPP** (0.259 mg) in equimolar quantities, which were added along with **pLeu-8Q** to the lipid solution. Lipid film was rehydrated with Millipore endotoxin-free water (900 μL) at 56 °C following extrusion with a 200 nm pore-size polycarbonate membrane. Finally, PBS (100 μL, 10×) was added to the formulation, producing **pLeu-8Qm** (1 mg/mL) in unilamellar liposomal formulations.

**Formulation of L2CPP**

The liposomes bearing targeting moieties were formulated in a similar manner to **L2**, except DC-targeting moieties CPP (0.25 mg)—added along with **pLeu-8Q** to the lipid solution. In addition, DDAB was excluded from lipid solution. Lipid film was rehydrated with 900 μL of Millipore endotoxin-free water at 56 °C and not extruded. Finally, 100 μL of 10× PBS was added to the formulation, producing the final concentration of **pLeu-8Qm** (1 mg/mL) as multilamellar liposomes.

### 2.4. Characterization of Nanoliposomes

Particle size, zeta potential and polydispersity of the vaccine candidates were measured by dynamic light scattering (DLS) using a Zetasizer (Nano ZX, Marvern, UK). Measurements were taken at 25 °C and 173° light scattering. DLS measurements were taken using a Zetasizer Nano ZP instrument (Malvern Instrument, UK) with Malvern Zetasizer Analyzer 6.2 software. Particle morphology was examined using transmission electron microscopy (TEM) (HT7700 Exalens, HITACHI Ltd., Tokyo, Japan) after vacuum drying. Briefly, samples were diluted in pure distilled water (1:100), dropped directly onto glow-discharged carbon-coated copper grids, then stained with uranyl acetate (2%). Samples were observed at 200.0 k× magnification.

### 2.5. Mice and TC-1 Cell Lines

TC-1 cells (murine C57BL/6 lung epithelial cells transformed with HPV-16 E6/E7 and ras oncogenes) were obtained from TC Wu, John Hopkins Cervical Cancer Research Lab, USA. TC-1 cells were cultured and maintained at 37 °C/5% CO_2_ in RPMI 1640 medium supplemented with 10% heat-inactivated fetal bovine serum. Female C57BL/6 (6–8-week-old) mice were purchased from the Animal Resources Centre (Perth, Western Australia). Animal experiments were approved by The University of Queensland Animal Ethics Committee (UQDI/TRI/351/15 and UQDI/032/20) and were carried out in accordance with National Health and Medical Research Council (NHMRC) of Australia guidelines.

### 2.6. In Vivo Tumor Treatment Experiments

C57BL/6 Female mice (8 mice per group in pilot study, and 6 mice per group in the second study) were challenged subcutaneously in the flank with 2 × 10^5^ TC-1 tumor cells/mouse (i.e., 1 × 10^5^ TC-1 tumor cells/side). After 7 days (pilot) or 11 days (second study), mice were injected in the flank with the vaccine candidates. Each mouse received peptide (100 μL) or liposome formulation containing antigen (100 μg). Mice also received PBS (100 µL, 1×) as a negative control. **L1** or a formulation containing **8Qm** (100 µg) and CpG ODN 1826 adjuvant (20 µg) dissolved in PBS were used as a positive control. Each mouse received a single dose of the vaccine/control solution. Tumor size was measured (by palpation and calipers) and recorded for each mouse every 2 days. Tumor volume was calculated using the formula: V (cm^3^) = 3.14 × [largest diameter × (perpendicular diameter)2]/6. The average tumor size per group (n = 8 or 6) was also calculated. Mice were euthanized when tumors reached 1 to 3 cm or if they started bleeding to avoid unnecessary suffering. An additional cohort of C57BL/6 mice (6 mice per group) were immunized with antigens, as described above. Mouse spleens were collected from these animals 10 days post immunization and used for the ELISPOT assay.

### 2.7. IFN-Gamma ELISPOT Assays

ELISPOT plates were coated with 5 μg/mL IFN-γ capture antibody (clone 14-7313-85 eBioscience, San Diego, CA, USA) in PBS at 4 °C overnight. The plates were then blocked with RPM/20% fetal bovine serum at room temperature for 3 h. Splenocytes from C57BL/6 mice were harvested from the spleens of naïve and immunized mice. The red blood cells were depleted using red blood cell lysing buffer (0.155 M ammonium chloride in 0.01 M Tris-HCl buffer, Sigma). Splenocytes were then resuspended in RPMI (Sigma) supplemented with 20% FACs (100 U/mL penicillin, 100 μg/mL streptomycin and 50 μM β-mercaptoethanol). The cells were plated at 5 × 10^5^ cells per well in triplicate on ELISPOT plates. The E7 peptide (10 μg/mL) was added alongside 10 ng/mL rhIL-2 to a final volume of 200 μL per well. Plates were incubated for 8 h at 37 °C and washed. Biotinylated IFN-γ detection antibody (2 μg/mL; clone R4-6A2; eBioscience) in BSA (1%) was then added at room temperature. The plates were washed, and bound cytokine was visualized with 3-amino-9-ethylcarbozole. Spots were counted with an ELISPOT reader. 

### 2.8. Statistical Analysis

All data were analyzed using GraphPad Prism 7 software. Kaplan−Meier survival curves were used to compare tumor treatment candidates. Differences in survival treatments were determined using the log-rank (Mantel–Cox) test, with *p* < 0.05 considered statistically significant. ELISPOT statistical analysis was performed using a one-way analysis of variance (ANOVA) followed by Tukey’s multiple comparisons test (ns, *p* > 0.05; *, *p* < 0.05).

## 3. Results and Discussion

### 3.1. Vaccine Design

In our previous attempts to develop efficient vaccine delivery systems, we have demonstrated that conjugation of the polyleucine moiety to a peptide antigen greatly enhanced antigen-specific humoral immune responses [20,21,23,24,25,26,27]. We also found that the polymer–antigen conjugate **D-8Qm** once formulated into liposomes (**L1**) was able to eradicate, to some extent, seven-days-old cancer in mice [27]. Herein, we have replaced the polyacrylate dendrimer (D) with polyleucine to produce the self-assembling conjugate, **pLeu-8Qm**. In addition, we have also combined the polyleucine strategy with a liposomal formulation applied for **D-8Qm** (**L1**) to produce **L2**. To further improve vaccine efficacy, we anchored a variety of DC-targeting moieties to **L2** liposomes (Figure 1), as antigen uptake by DCs (and other antigen presenting cells) is a key process in initializing natural immune responses, including anticancer cellular immunity [33]. All the targeting moieties have been lipidated to allow their anchoring to liposomes. Here, lipopeptides **D1** and **D2** were bearing DCpep-1 and DCpep-2 epitopes, respectively, where both DCpep-1 and DCpep-2 are reported to have DC-targeting properties [34]. In addition, DCpep-1 has been shown to stimulate specific T-cell responses against tumors [35,36,37], while DCpep-2 regulated inflammatory mediators [38,39]. The incorporation of a mannose-based ligand into liposome formulations has been previously reported as being effective in promoting CTL responses [40,41,42]. We designed our ligands (**M1**–**M3**) to have different spacer lengths between the mannose and lipidic moieties, where the importance of these spacers has been previously reported [3,43,44,45]. We have also demonstrated the mechanism of mannose-receptor-mediated uptake [46]. Importantly, the ability of **M2** anchored into liposomes to greatly enhance humoral immune responses against a bacterial antigen has also been proven (unpublished data). Along the same line, we also demonstrated that the mannose–lipid conjugate **M3** easily anchored into liposomes induced cellular immunity against malaria and babesia parasites [31,47,48,49]. Finally, we had also previously proven that the lapidated-cell-penetrating peptide KALA (**CPP**), upon anchoring to liposomes, triggered the production of opsonic antibodies against a co-anchored peptide antigen [32]. The ability of cell-penetrating peptides to improve the cellular immunity of the co-delivered antigen has also been reported by other groups [50]. Identically produced cationic liposomes were used in all formulations (**L1**, **L2**, **L2DC1**, **L2DC2**, **L2M1**, **L2M2**, **L2M3**) except **L2CPP**, where neutral multilamellar liposomes were applied. Here, the use of cationic liposomes in combination with a **CPP** was redundant due to the very high cationic charge of the KALA peptide, and a **CPP** was more effective when combined with multilamellar (not unilamellar) liposomes [32].

### 3.2. Vaccine Preparation

All the peptide components of vaccines and targeting moieties were synthesized using Fmoc-SPPS. **pLeu-8Qm** was also produced as a single molecule using the standard SPPS methodology. **pLeu-8Qm** was incorporated in DC-targeting liposomal formulations and used for a pilot immunization study. The polymeric conjugate **D-8Qm** was produced by conjugation of the **8Qm** peptide to polyacrylate **D** by the copper-catalyzed alkyne−azide cycloaddition (CuAAC) reaction and self-assembled into particles by the solvent replacement method, as previously reported [19,51]. Dialysis was performed in water for three days to remove residual peptide, copper and organic solvents, and elemental analysis was used to confirm the formation of the product, which was in line with the methods of many previous studies [10,11,20,51,52], as the conjugate contained a higher nitrogen/carbon ratio compared with the polyacrylate alone. Theoretical and observed (nitrogen [N]/carbon [C]) ratios were used to calculate the exact substitution of the polymer core with the peptide epitopes. The observed nitrogen–carbon ratio for **D-8Qm** (N/C = 0.115) was higher than that of the polymer alone (N/C= 0.017) and corresponded to 83% of the substitution rate of the dendrimer with **8Qm** peptide (similar to that previously reported at 85% [19]). Both **D-8Qm** and **pLeu-8Qm**, as amphiphilic conjugates, were easily incorporated (anchored) into liposomes [19,21] to form **L1** and **L2**, respectively (Figure 1). 

DCs-targeting moieties, **DC1**, **DC2** and **M1**, were designed to anchor targeting moieties, DCpep1, DCpep2 and mannose, to liposomes by the DOPE lipid and PEG linker for optimal receptor recognition (Figure 1) [53]. First, DOPE-PEG3.4k-aklyne was synthesized using our published procedure (Supporting Information, Figure S2) [29] before being individually reacted with azide derivatives of DCpep1, DCpep2 and mannose to produce **DC1**, **DC2** and **M1**, respectively (Supporting Information, Figures S3 and S4). Other targeting moieties (**M2**, **M3** and **CPP**) were synthesized as reported [31,32]. The conjugates **D-8Qm and pLeu-8Qm,** as well as the lipidated targeting moieties (**DC1**, **DC2**, **M1**, **M2**, **M3**, **CPP**), were incorporated into the liposomal bilayer during a thin-film lipid formulation. Lipid films were hydrated and sonicated to produce **L1**, as previously reported [19], or extruded (**L2**, **L2DC1**, **L2DC2**, **L2M1**, **L2M2**, **L2M3**) with 200 nm pore membranes to form uniform-sized liposomes, except **L2CPP**, which was formulated as multilamellar liposomes (extrusion step was omitted during liposomes preparation). 

The size and surface charge of the vaccine candidates were analyzed using DLS (Table 1, Supporting Information, Figure S5). Both **D-8Qm** and **pLeu-8Qm** produced aggregates of sub/micron sizes with high polydispersity (polydispersity index; PDI = 0.6–0.7) and a similar negative charge (ζ = −10–−14 mV). In contrast, all liposome formulations were positively charged and relatively monodispersed, except **L1** and **L2CPP**. Unilamellar liposomes bearing **pLeu-8Qm** were produced in the size range of 150–200 nm, which was typically more immunogenic compared with larger-sized particles [54,55,56]. Multilamellar liposome **L2CPP** formed submicron particles (0.6 µm, PDI = 0.4) with a high positive charge. Additionally, the size and morphology of the vaccine candidates were analyzed by TEM (Supporting Information, Figure S6). Large particle aggregates were detected on images of **D-8Qm**, while all liposomes formed typical spherical liposome structures, which were uniform in size (100–250 nm) without visible traces of large aggregates, as detected by DLS in the case of **L1** and **L2DC1** (Supporting Information, Figure S5). 

### 3.3. Immunological Evaluation of the Vaccine Candidates

**Figure 2:**
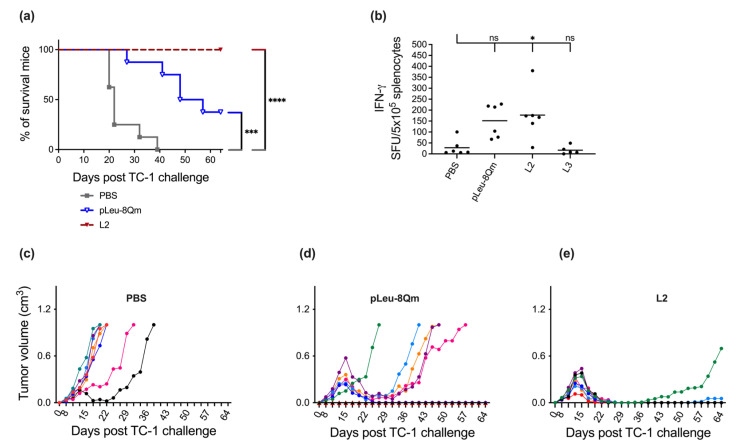
In vivo tumor treatment experiments and CD8+ T-cell activation. C57BL/6 mice (n = 8 mice/group) were inoculated subcutaneously with TC-1 tumor cells (day 0) and vaccinated with different immunogens on day 7. (**a**) Survival rate was monitored and time to death plotted on a Kaplan–Meier survival curve. Tumor volume was monitored and plotted for each individual mouse immunized with (**b**) PBS; (**c**) **pLeu-8Qm**; (**d**) **L2**. The survival rate of each group was compared with the negative control (PBS) and was analyzed using the log-rank (Mantel–Cox) test (*** *p* < 0.001; **** *p* < 0.0001). (**e**) Assessment of CD8+ T-cell responses in mice subcutaneously immunized with the vaccine candidates. Mouse spleens were harvested ten days after vaccination and IFN-γ production in response to short E749-57 (RAHYNIVTF) CD8 peptide epitope was determined by ELISPOT (n = 6 mice/group). The **L3** group (irrelevant control) was identical to **L2** except the antigen **8Qm** was replaced with J8 B-cell epitope derived from group A streptococcus (unrelated epitope). ELIPOST data were pooled from two independent experiments and analyzed using one-way ANOVA followed by Tukey’s post hoc multiple comparison test (ns, *p* > 0.05; *, *p* < 0.05).

At first, the therapeutic effect of the vaccine candidates on established tumors was evaluated in eight-week-old female C57BL/6 mice seven days post TC-1 tumor implantation (Figure 2). Average tumor growth was fast, with only 25% of the negative control (PBS-treated) mice surviving to day 21. Alone, **pLeu-8Qm** decreased tumor growth and prolonged mouse survival. The incorporation of **pLeu-8Qm** into liposomes (**L2**) greatly improved vaccine efficacy. While all mice treated with **L2** survived 64 days, two of the eight mice had tumor growth recurrence after days 34 and 56. The improved ability of the liposome-based **L2** vaccine candidate in triggering an antitumor immune response compared with **pLeu-8Qm** alone might be attributed to the size of the particles. **pLeu-8Qm** formed highly polydisperse submicroparticles, while **L2** formed nanoparticles (<200 nm); however, smaller particles are widely reported to be more immunogenic [57,58].

As the vaccine candidates investigated in this study aimed to stimulate CD8+ CTLs in order to eradicate HPV-infected cells, the recall of IFN-γ production by CD8+ T-cells was examined in immunized mice in response to MHC classIrestricted E7 peptide re-stimulation by ELISPOT (Figure 2). The immunization of mice with **pLeu-8Qm** delivered by liposomes induced significantly higher levels of IFN-γ compared with PBS, corresponding with its antitumor potency. This demonstrated that the downstream priming of the T-cells via INF-γ activation led to the eradication of tumors following subcutaneous immunization. Moreover, this antitumor efficacy was achieved without the presence of an external adjuvant, assistance of chemotherapy, multiple immunizations and/or boosting with immune checkpoint inhibitors [59,60,61,62].

**Figure 3:**
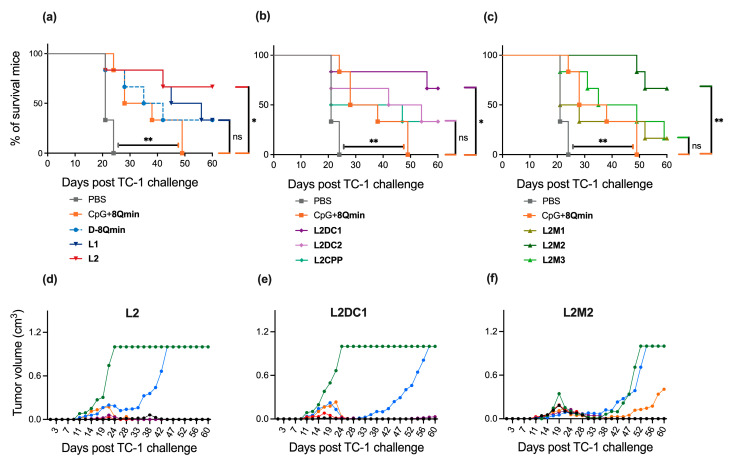
In vivo tumor treatment experiments. C57BL/6 mice (n = 6 mice/group) were inoculated subcutaneously with TC-1 tumor cells (day 0) and vaccinated with different immunogens on day 11. (**a**–**c**) Survival rate was monitored and time to death plotted on a Kaplan–Meier survival curve (separated here into three panels for clarity). The survival rate of each group was compared with the positive control (CpG + **8Qm**) and was analyzed using the log-rank (Mantel–Cox) test (ns, *p* > 0.05; *, *p* < 0.05; ** *p* < 0.01). Tumor volume was monitored and plotted for each individual mouse immunized with (**d**) **L2**; (**e**) **L2DC1**; (**f**) **L2M2**. Tumor volume charts for other groups are presented in Supporting Information, Figure S7.

Encouraged by the outcomes of the pilot study, we designed several new vaccine candidates (Figure 1) and examined them under more demanding conditions (Figure 3, Supporting Information, Figure S7). The efficacy of the vaccine candidates was evaluated by testing against 11-day-old tumors following a single immunization to demonstrate the superiority of the strategy compared with our previous studies. These previous studies utilized 8Qm-based peptide–polymer conjugate vaccines targeting 3-day-old smaller tumors [11], a prime-boost immunization schedule using HPV E6- and E7-epitope-based multivalent conjugates with a booster [18], or the use of 8Qm emulsified in the commercialized adjuvant, Montanide ISA 51, to achieve significant antitumor effects [10]. Previously examined polyacrylate-based vaccine **D-8Qm** and its liposomal formulation L1 have also been assessed. In addition, the classical “cellular” adjuvant, CpG, was used as a positive control. CpG has been recently extensively investigated as an effective immune stimulator in the development of therapeutic vaccines against HPV-related cancers [4,7,63,64,65]. Three vaccine candidates, **L2**, **L2DC1** and **L2M2,** delayed tumor regrowth and induced tumor regression with the highest survival rate (67% on day 60). All surviving mice treated with **L2** achieved complete tumor regression on day 60, while full tumor regression was observed in three out of six mice in both **L2DC1** and **L2M2** (Figure 3). Moreover, **L2M2** induced some tumor regression in all mice, and these mice survived up to day 47 following a single immunization. Importantly, all three vaccines (**L2**, **L2DC1** and **L2M2**) were significantly more effective as therapeutic vaccines than **8Qm** adjuvanted with CpG. Mice immunized with **8Qm**/CpG showed a delay in tumor growth compared with the negative control (PBS); however, they ultimately had the same survival rate of 0% on day 60. Among the three leading formulations, even the simple liposome formulation **L2** was more effective at inducing tumor regression than the previous leading vaccine candidates, **D-8Qm** and **L1** [19]. It is also worth noting that when unconjugated and adjuvant-free 8Qm was incorporated into the same liposomal formulation, therapeutic potential was not observed [19]. Other DC-targeting formulations (**L2DC2**, **L2M1**, **L2M3** and **L2CPP**) were less effective than the untargeted **L2** formulation and not significantly more effective than **8Qm**/CpG. In addition, there was no correlation between the size of liposome particles and vaccine efficacy. The leading vaccine candidates, **L2**, **L2DC1** and **L2M2**, had particle sizes in similar ranges to other liposomal formulations, except **L2CPP** (Table 1). 

Notably, targeting moieties did not improve the final survival rate; however, the formulation bearing lipidated mannose (**M2**) delayed tumor growth more significantly than other formulations. Thus, further investigations on this formulation are required. For example, the anticancer efficacy of **L2M2** might be greatly enhanced by the administration of a boosting dose around 3–4 weeks following the primary vaccination. 

In our study, mice immunized with **L2**, **L2DC1** and **L2M2** demonstrated improved survival 60 days after the inoculation of the same density of TC1 tumor cells (1 × 10^5^), despite the more advanced stage of tumor development at the time of the initiation of vaccine treatment (11 days post tumor cell inoculation versus 7 or 8 days in other studies [66,67]). Furthermore, within the framework of a single immunization schedule, the **L2**-, **L2DC1**- and **L2M2**-immunized mice displayed tumor eradication in some cases, outperforming prior studies utilizing multiple immunizations, which failed to achieve complete tumor regression [66,67,68]. Additionally, the efficacy of our vaccine formulation was maintained without the use of multiepitopes from both E6 and E7 proteins [69]; the addition of adjuvants, such as flagellin [70], Montanide ISA 720 [71] or a combination of poly I:C and CpG [63]; or synergistic therapy with immune checkpoint inhibitors, such as anti-CTLA4 [72] or anti-PD1 [73].

## 4. Conclusions

We demonstrated that a fully defined, natural, hydrophobic amino-acid-based polymer conjugated to peptide antigen acts as an efficient vaccine delivery system. Importantly, polyleucine mimics the transmembrane fragments of proteins, allowing the anchoring of the conjugate to liposomes and effectively triggering cellular immunity to destroy tumor cells following a single immunization without the use of an additional adjuvant. In contrast with nanotechnology-based strategies, which usually offer incompletely defined systems, our strategy utilizes a polymer built from natural amino acids that is both a single molecule and a single isomer. Given the need for the vaccine treatment of intracellular infectious diseases and malignancies, we anticipate numerous applications for the vaccine delivery system presented here.

## Figures and Tables

**Table 1:** Physicochemical characterization of vaccine candidates.

Vaccine	Targeting Moiety	Size (nm ± SD)	PDI	Charge (mV ± SD)
Blank liposomes	-	165 ± 4	0.06 ± 0.01	61 ± 2
**D-8Qm**	-	250 ± 50; 1500 ± 100	0.70 ± 0.06	−14 ± 1
**pLeu-8Qm**	-	750 ± 350	0.60 ± 0.10	−10 ± 5
**L1**	-	320 ± 20; 4500 ± 600	0.50 ± 0.05	28 ± 2
**L2**	-	157 ± 2	0.10 ± 0.01	24 ± 2
**L2DC1**	**DC1**	200 ± 10; 3200 ± 1100	0.30 ± 0.01	17 ± 2
**L2DC2**	**DC2**	160 ± 10	0.05 ± 0.01	23 ± 2
**L2M1**	**M1**	162 ± 6	0.10 ± 0.01	13 ± 1
**L2M2**	**M2**	174 ± 2; 4800 ± 300	0.20 ± 0.01	20 ± 1
**L2M3**	**M3**	143 ± 2	0.05 ± 0.01	23 ± 2
**L2CPP ^1^**	**CPP**	610 ± 60	0.40 ± 0.03	54 ± 5

^1^ Liposome formulation **L2CPP** did not include the cationic lipid (DDAB) and was not extruded.

## Data Availability

Not applicable.

## Supplementary Materials

The following supporting information can be downloaded at: https://www.mdpi.com/article/10.3390/pharmaceutics15020602/s1, Figure S1: ESI-MS and HPLC trace of **pLeu-8Qm**. Figure S2: Synthesis of compound DOPE-PEG3.4k-alkyne (**4**). **Figure S3**: The MALDI-TOF mass spectrometry of **DC1** and **DC2**. Figure S4: The MALDI-TOF mass spectrometry of DOPE-PEG3.4k-alkyne (**4**) and DOPE-PEG3400-mannose (**M1**). Figure S5: Particles size of (A) **D-8Qm**, (B) **pLeu-8Qm,** (C) **L1**, (D) **L2**, (E) **L2DC1**, (F) **L2DC2**, (G) **L2M1**, (H) **L2M2**, (I) **L2M3** and (J) **L2CPP** were recorded by dynamic light scattering (DLS). Figure S6: Transmission electron microscopy images of (A) **D-8Qm**, (B) **L1**, (C) **L2**, (D) **L2DC1**, (E) **L2DC2**, (F) **L2M1**, (G) **L2M2**, (H) **L2M3** and (I) **L2CPP**. Figure S7: In vivo tumor treatment experiments. Tumor volume was monitored and plotted for each individual mouse immunized with **PBS,** CpG **+ 8Qm**, **D8Qm**, **L1**, **L2DC2**, **L2M1**, **L2M3**, and **L2CPP**.
