# Supplementary material for: Liposomal Formulations of a Polyleucine–Antigen Conjugate as Therapeutic Vaccines against Cervical Cancer
Source: Pharmaceutics. 2023 Feb 10;15(2):602. doi: 10.3390/pharmaceutics15020602 (PMC9965676; doi:10.3390/pharmaceutics15020602)
Supplement: Supplementary file 1 [file pharmaceutics-15-00602-s001.zip › pharmaceutics-2186985-supplementary.pdf]

Supporting Information

Liposomal formulations of a polyleucine-antigen conjugate as therapeutic vaccines against cervical cancer.

Farrhana Z. Firdaus[a], Stacey Bartlett[a], Waleed M. Hussein[a], Lantian Lu[a], Quentin Wright[b], Wenbin Huang[a], Ummey J. Nahar[a], Jieru Yang[a], Mattaka Khongkow[a,c], Margaret Veitch[b], Prashamsa Koirala[a], Uracha R. Ruktanonchai[c], Michael J. Monteiro[d], Jazmina G. Cruz[b], Rachel J. Stephenson[a], James W. Wells[b], Istvan Toth[a,e,*], Mariusz Skwarczynski[a,*]

[1] *The University of Queensland, School of Chemistry and Molecular Biosciences, St Lucia QLD 4072, Australia*

[2] *The University of Queensland Diamantina Institute, The University of Queensland, Translational Research Institute, Brisbane, Australia.*

[3] *National Nanotechnology Center (NANOTEC), National Science and Technology Development Agency, 111 Thailand Science Park, Phahonyothin Rd., Khlong Luang, Pathumthani 12120, Thailand.*

[4]*Australian Institute for Bioengineering and Nanotechnology, The University of Queensland, Brisbane, QLD 4072, Australia*

[5] *The University of Queensland, School of Pharmacy, Woolloongabba, QLD 4102, Australia*

[6] *The University of Queensland, Institute for Molecular Biosciences, St Lucia, QLD 4072, Australia*

Corresponding Author:

Istvan Toth i.toth@uq.edu.au

Mariusz Skwarczynski m.skwarczynski@uq.edu.au

A)

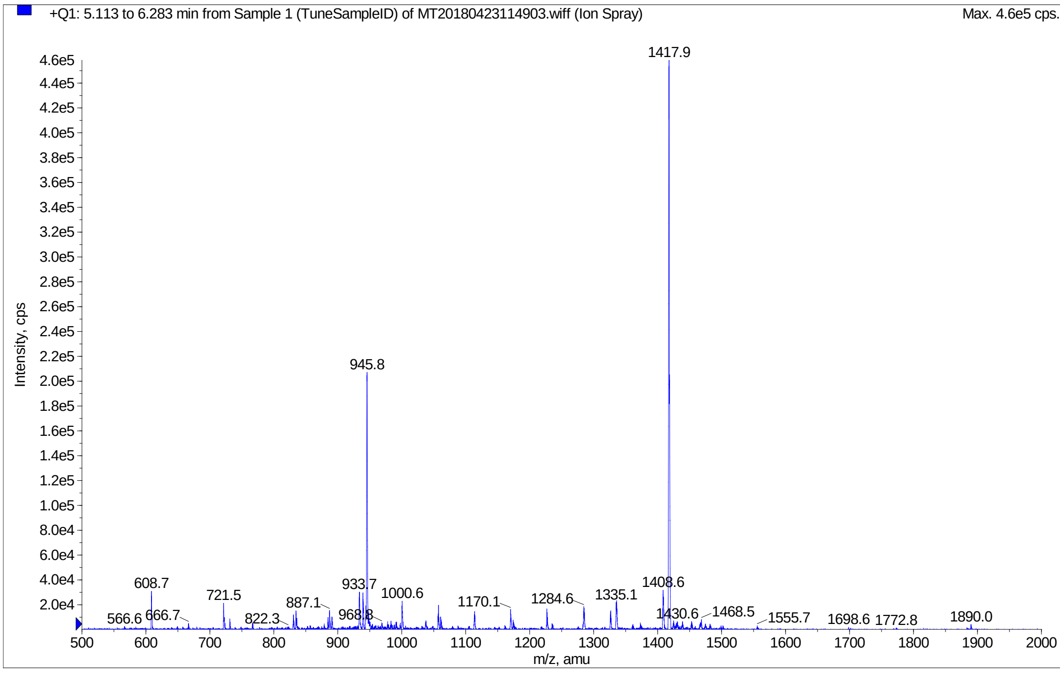

B)

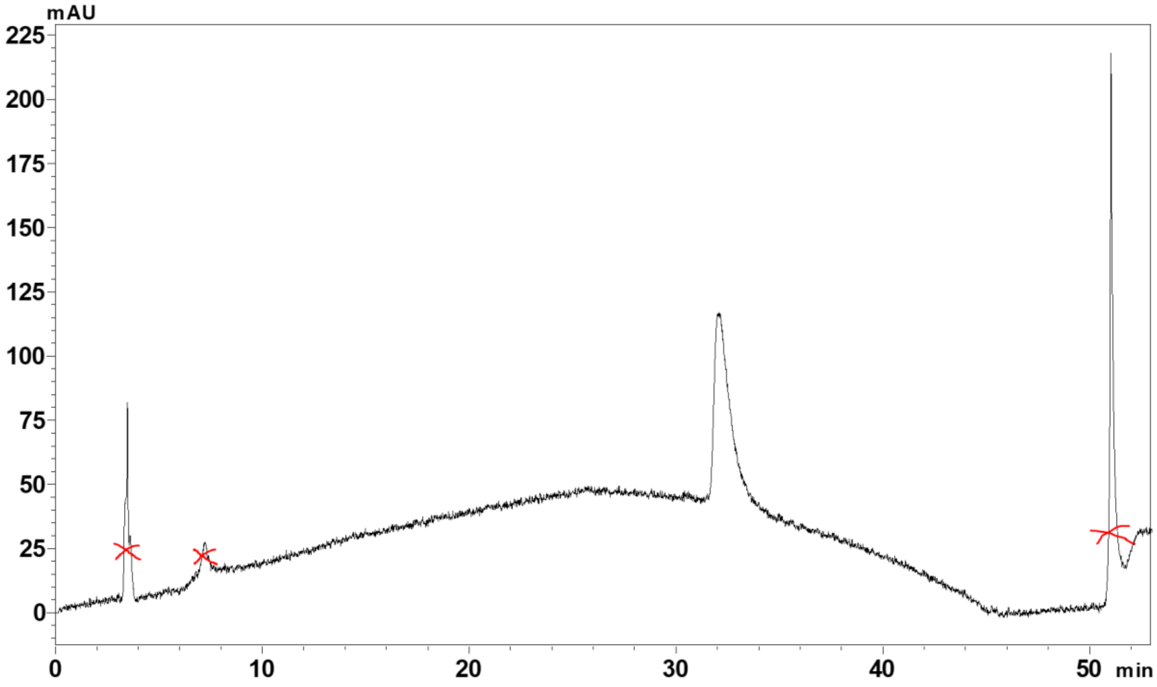

Figure S1. (A) ESI-MS: *m/z* 1417.7 (calculated 1417.2) $[M+2H]^{2+}$; 945.7 (calculated 945.2) $[M+3H]^{3+}$; MW = 2832.5; and (B) HPLC trace of **pLeu-8Qm**. t_R= 32 min.

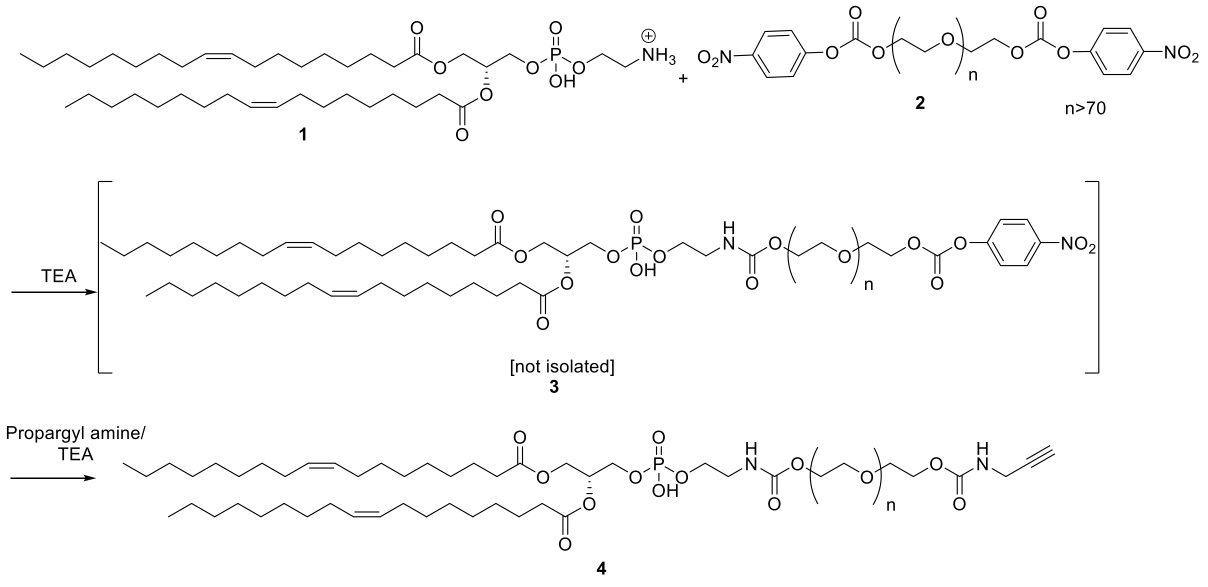

Figure S2. Synthesis of compound DOPE-PEG3.4k-alkyne (**4**) as per published procedure.[1]

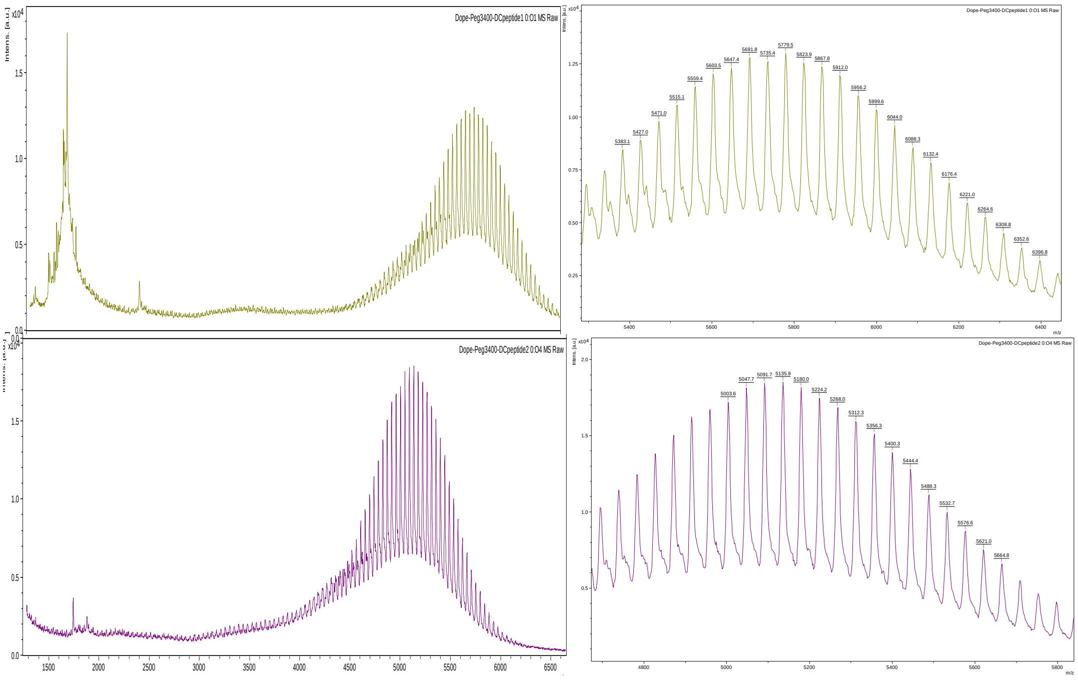

Figure S3. The MALDI-TOF mass spectrometry of **DC1** (upper panel) and **DC2** (lower panel).

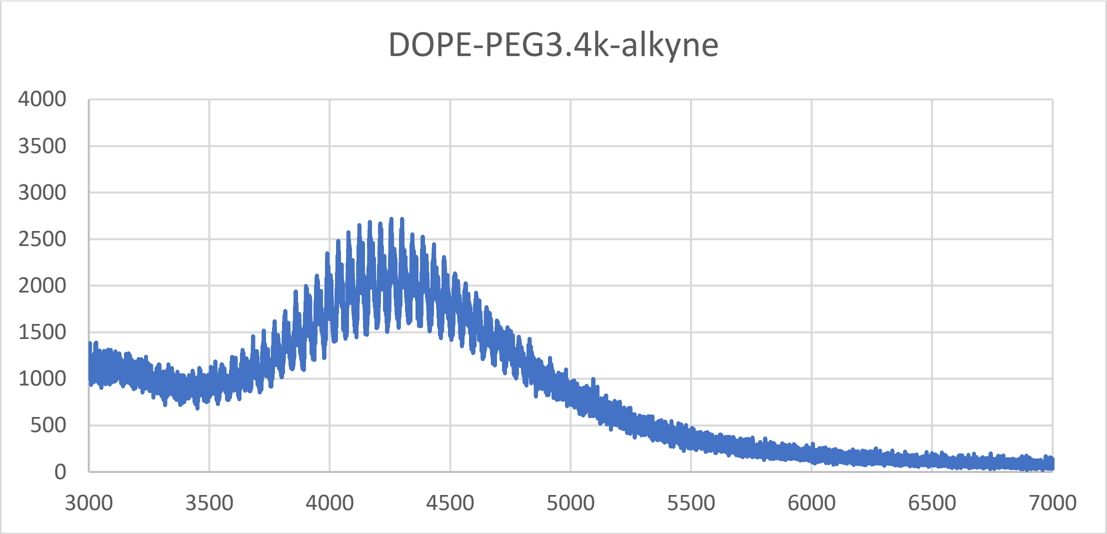

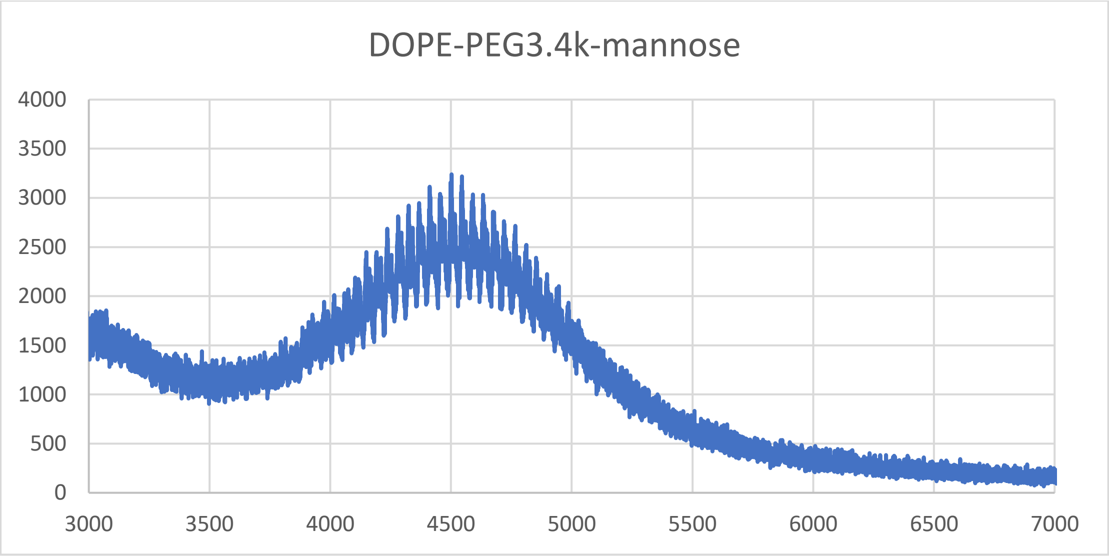

Figure S4. The MALDI-TOF mass spectrometry of Top = DOPE-PEG3.4k-alkyne (**4**), Bottom = DOPE-PEG3400-mannose (**M1**)

A)

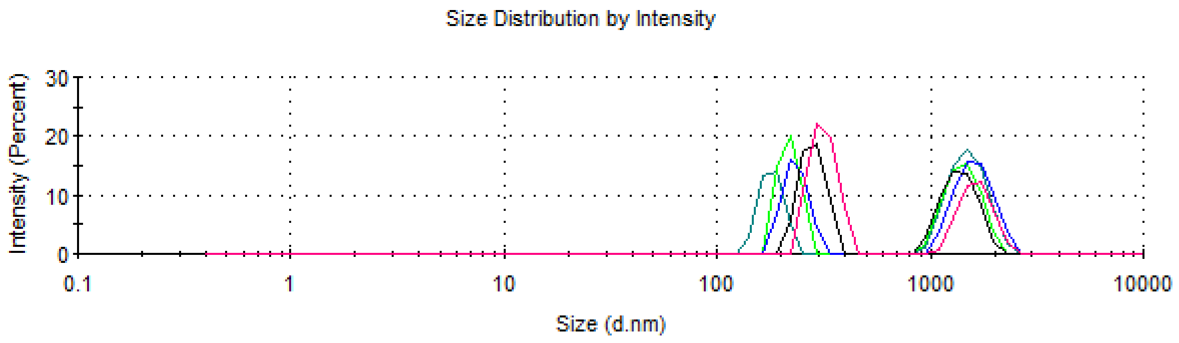
Size Distribution by Intensity
Intensity (Percent)
30
20
10
0
0.1
1
10
100
1000
10000
Size (d.nm)

B)

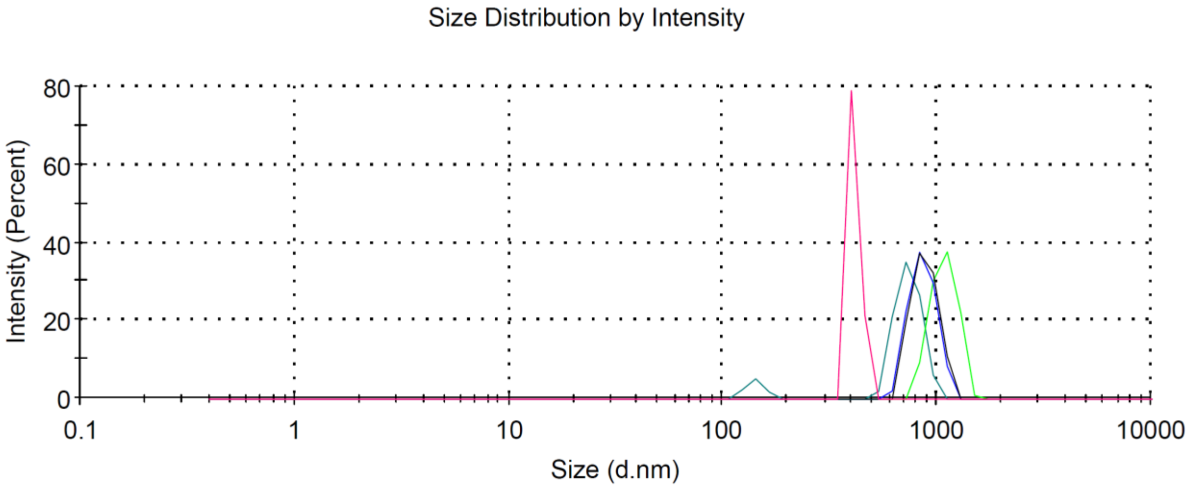
Size Distribution by Intensity
Intensity (Percent)
80
60
40
20
0
0.1
1
10
100
1000
10000
Size (d.nm)

C)

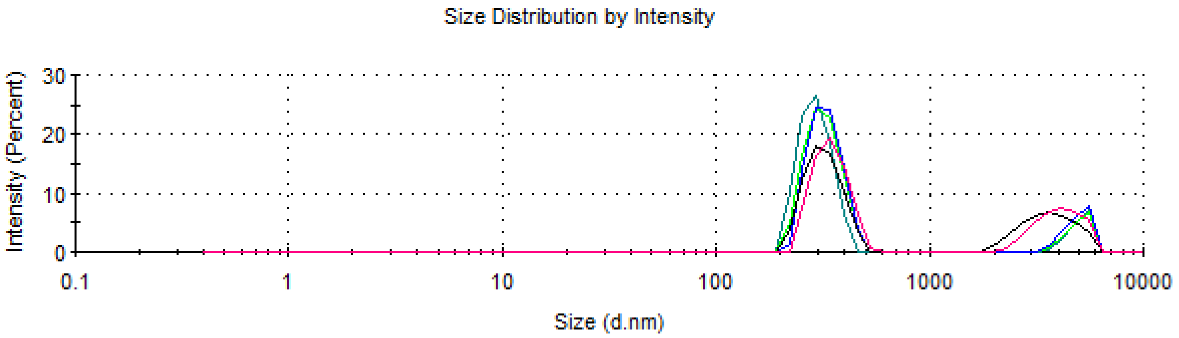
Size Distribution by Intensity
Intensity (Percent)
30
20
10
0
0.1
1
10
100
1000
10000
Size (d.nm)

D)

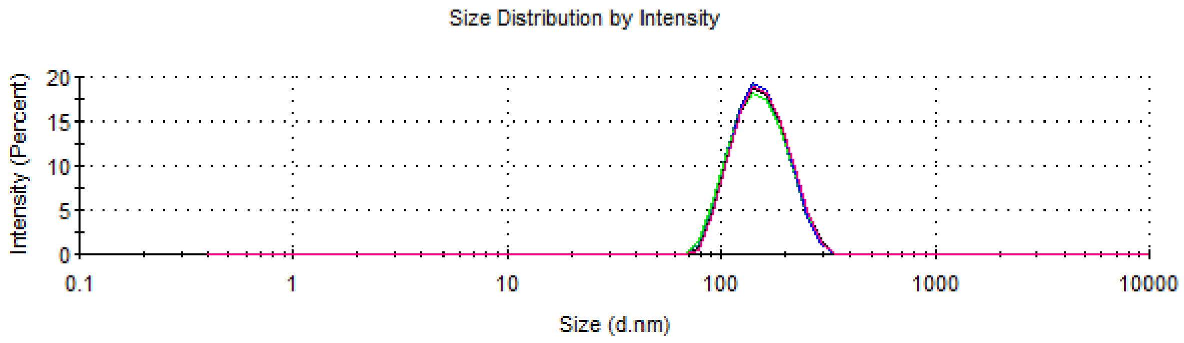
Size Distribution by Intensity
Intensity (Percent)
20
15
10
5
0
0.1
1
10
100
1000
10000
Size (d.nm)

E)

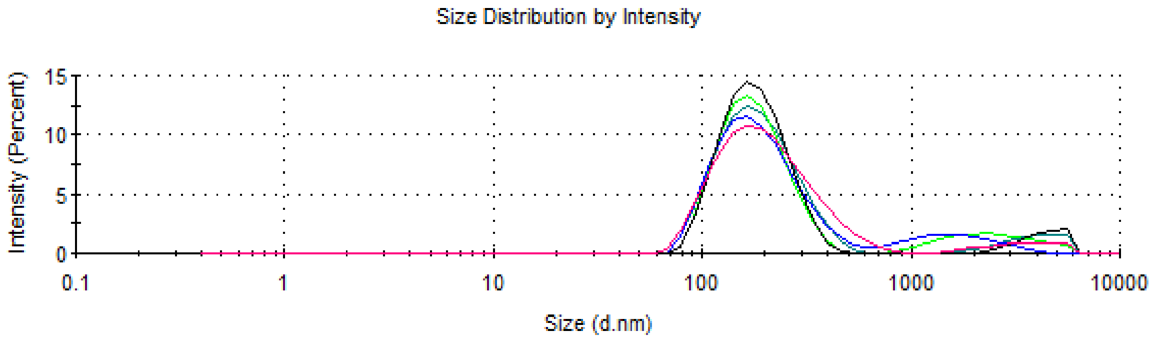
Size Distribution by Intensity
Intensity (Percent)
15
10
5
0
0.1
1
10
100
1000
10000
Size (d.nm)

F)

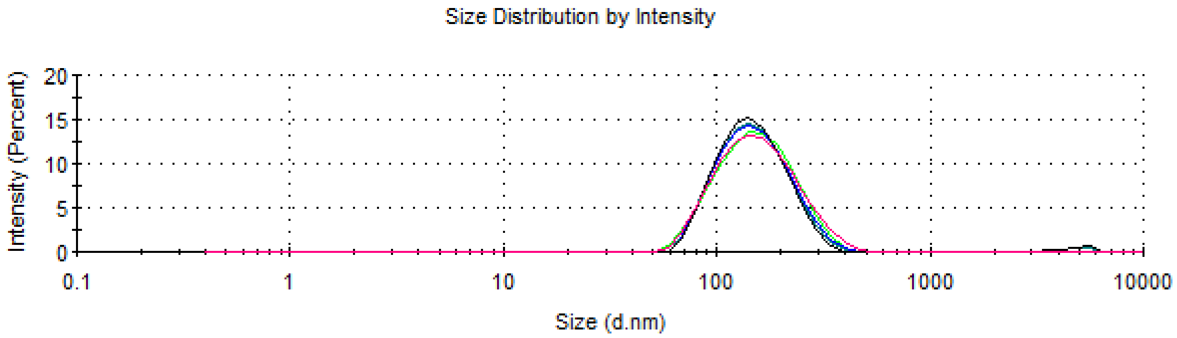
Size Distribution by Intensity
Intensity (Percent)
20
15
10
5
0
0.1
1
10
100
1000
10000
Size (d.nm)

G)

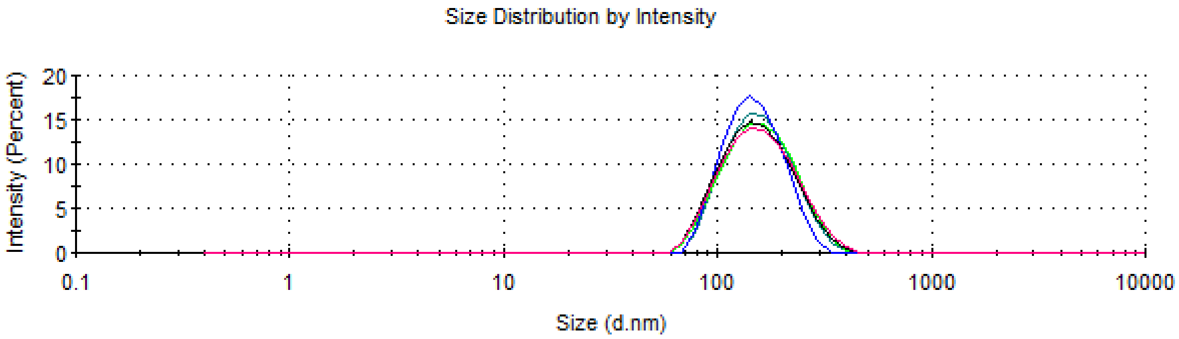
Size Distribution by Intensity
Intensity (Percent)
20
15
10
5
0
0.1
1
10
100
1000
10000
Size (d.nm)

H)

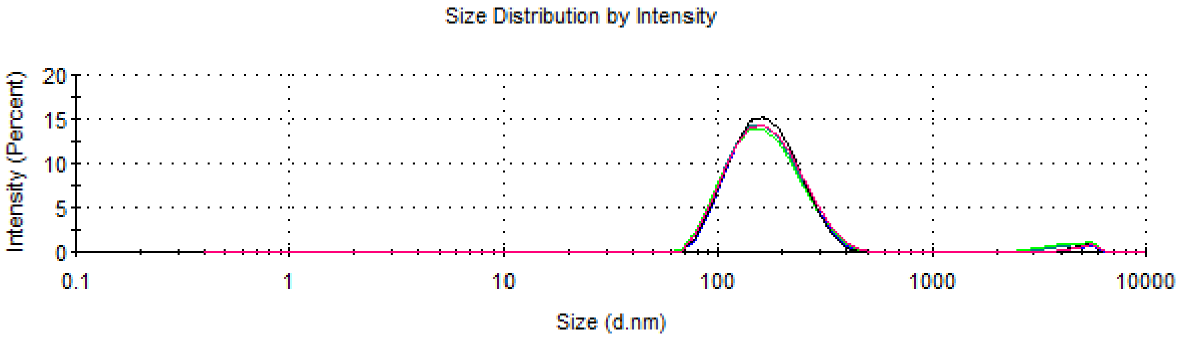
Size Distribution by Intensity
Intensity (Percent)
20
15
10
5
0
0.1
1
10
100
1000
10000
Size (d.nm)

I)

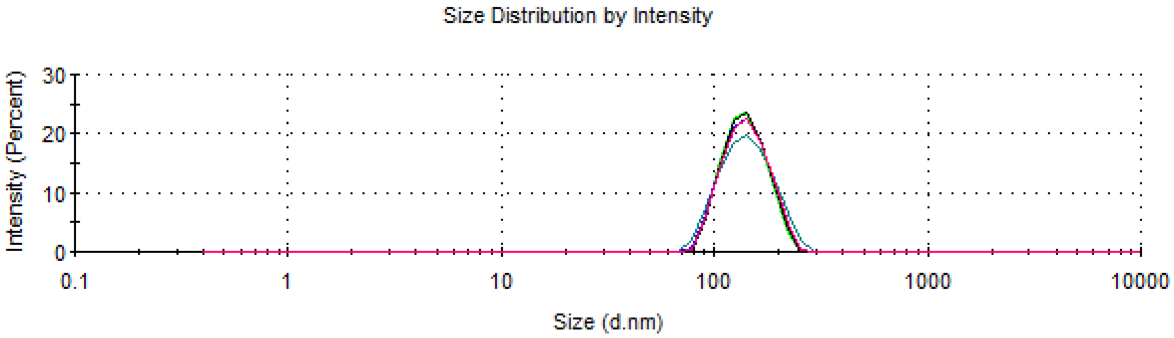

J)

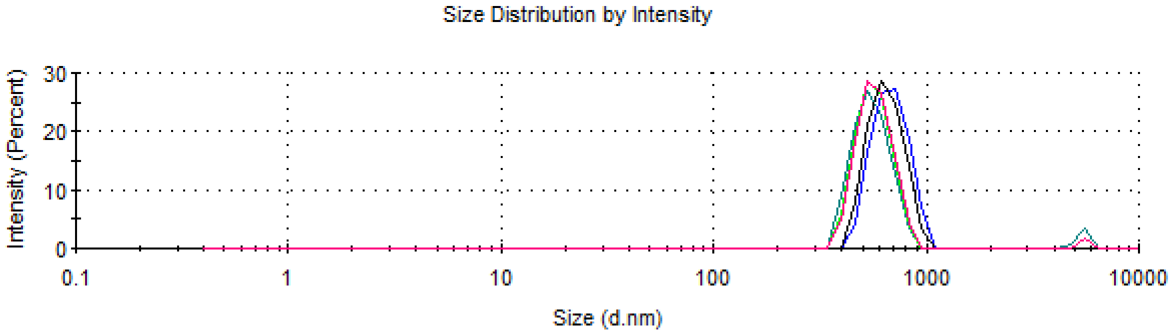

Figure S5. Particles size of A) **D-8Qm**, B) **pLeu-8Qm,** C) **L1**, D) **L2**, E) **L2DC1**, F) **L2DC2**, G) **L2M1**, H) **L2M2**, I) **L2M3**, and J) **L2CPP**. Five independent measurements per compound were recorded by dynamic light scattering (DLS).

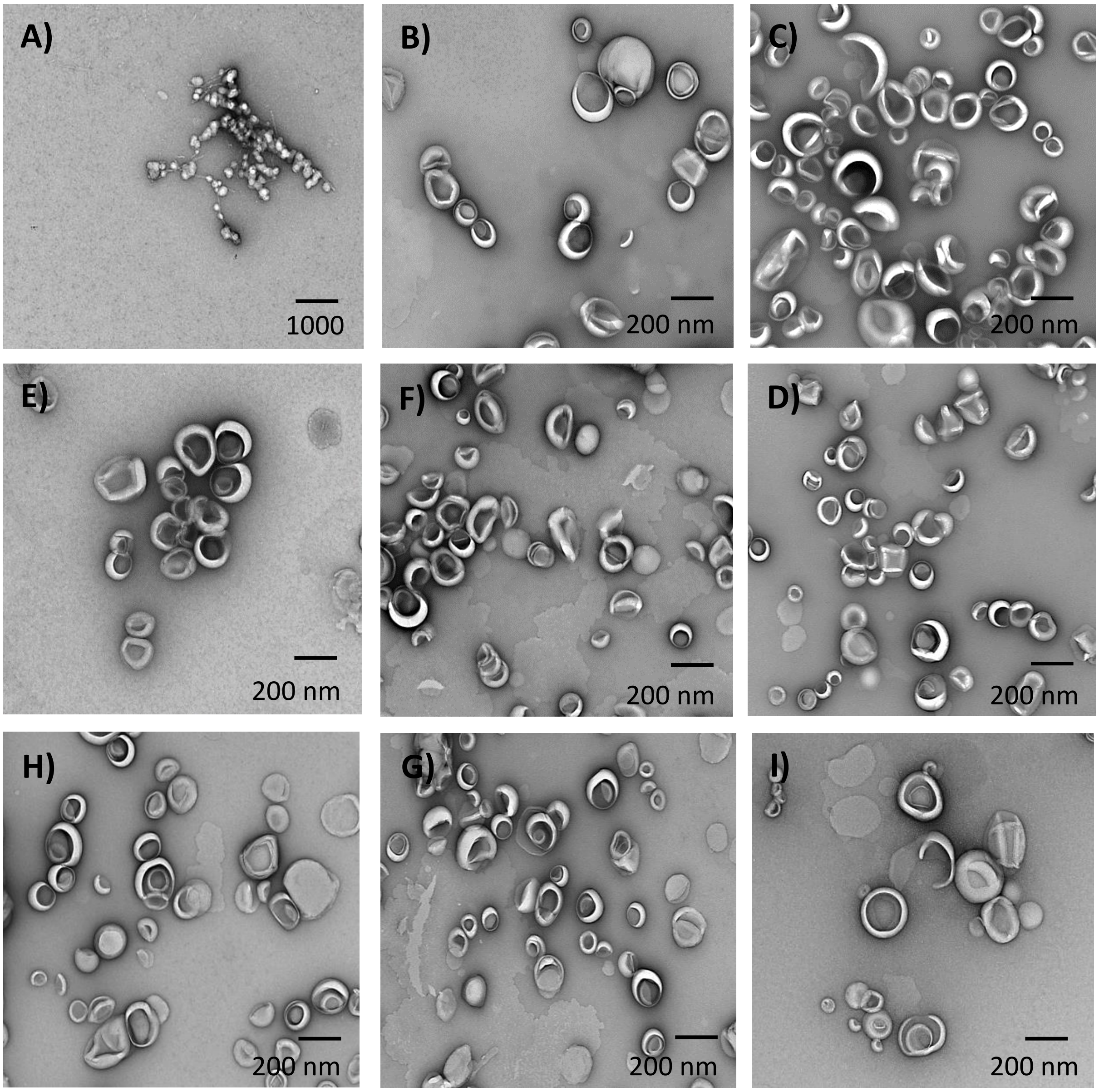

Figure S6. Transmission electron microscopy images of A) **D-8Qm**, B) **L1**, C) **L2**, D) **L2DC1**, E) **L2DC2**, F) **L2M1**, G) **L2M2**, H) **L2M3**, and I) **L2CPP** stained with 2% uranyl acetate (bar = 200 and 1000 nm).

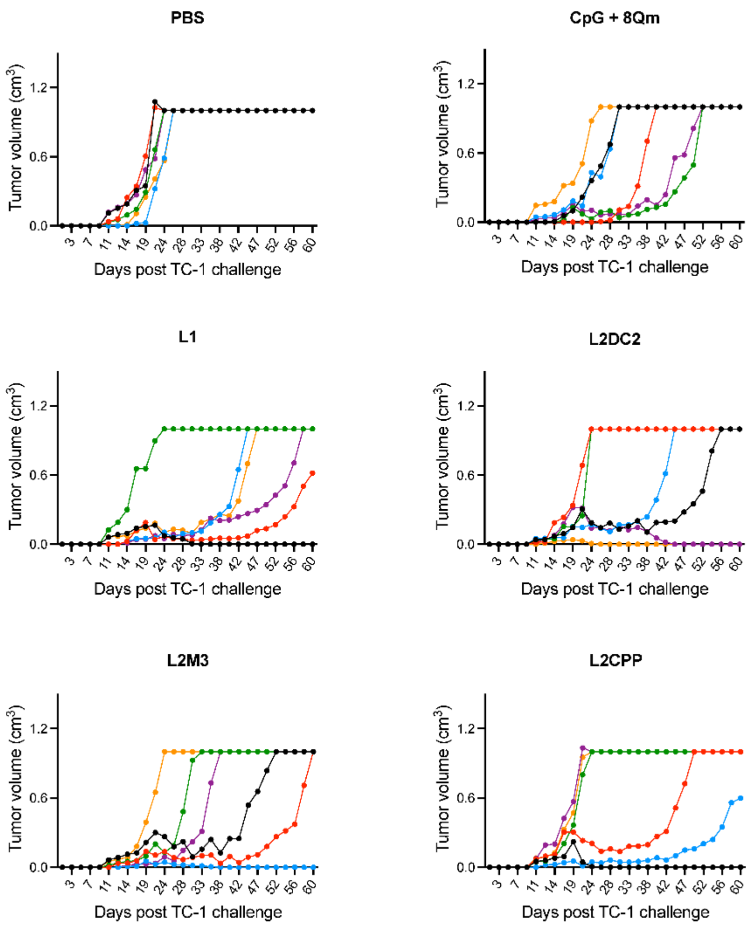

Figure S7. *In vivo* tumour treatment experiments. C57BL/6 mice (n = 8 mice/group) were inoculated subcutaneously with TC-1 tumour cells (day 0) and vaccinated with different immunogens on day 11. Tumour volume was monitored and plotted for each individual mouse immunised with **PBS, CpG + 8Qm**, **D8Qm**, **L1**, **L2DC2**, **L2M1**, **L2M3**, and **L2CPP**.

References

1. Hussein, W. M.; Cheong, Y. S.; Liu, C.; Liu, G.; Begum, A. A.; Attallah, M. A.; Moyle, P. M.; Torchilin, V.; Smith, R.; Toth, I. Peptide-based targeted polymeric nanoparticles for siRNA delivery. *Nanotechnology* **2019,** 30.
